# The Future Evolution of the Mortality Acceleration Due to the COVID-19: The Charlson Comorbidity Index in Stochastic Setting

**DOI:** 10.3389/fcvm.2022.938086

**Published:** 2022-07-14

**Authors:** Maria Carannante, Valeria D'Amato, Guido Iaccarino

**Affiliations:** ^1^Department of Pharmacy, University of Salerno, Fisciano, Italy; ^2^Department of Advanced Biomedical Sciences, University of Naples Federico II, Naples, Italy

**Keywords:** mortality projections, Charlson Comorbidity Index, relative frailty, proportional hazards model, stochastic modeling

## Abstract

The empirical evidence from different countries point out many of those who die from coronavirus would have died anyway in the relatively near future due to their existing frailties or co-morbidities. The *acceleration* of the mortality conceives the underlying insight according to deaths are “accelerated” ahead of schedule due to COVID-19. Starting from this idea, we forecast the future mortality acceleration, based on the deterioration due to the presence of the comorbidities at COVID-19 diagnosis. Accordingly, we explicitly determine the contribution of each comorbidity on the acceleration forecasting, showing the future trend of the excess of deaths due to the COVID-19. To this aim, our proposal consists in developing a revised Charlson Comorbidity Index in a stochastic environment. Based on a post-stratification scheme, we obtain an unbiased comorbidity index that varies by age, centered on the reference population.

## 1. Introduction

The ongoing outbreak of the novel coronavirus disease (COVID-19) was announced on the 11th of March 2020 by the General Director of the World Health Organization (1).

Since the emergence, the governments, the healthcare systems and the whole international community tried to address critical gaps in knowledge and response and readiness tools and activities. The scientific literature developed several methodologies based on different approaches, assumptions, range of predictions and metrics about the major findings as to the virus, the outbreak, transmission dynamics, disease progression and severity.

Forecasting COVID-19 mortality plays a key role in many fields such as healthcare, to ensure readiness to provide clinical care with early identification protocols, international guidelines as well as the economic system, to build a more resilient society, avoiding disruptive financial consequences on the economic sectors.

Some authors discuss “how will the temporarily stressed mortality rates change the post-COVID-19 mortality rates” (2), by introducing the concept of mortality shocks. The mortality changes due to the pandemic have been described as a shift in the apparent age (3). In general, as argued by Milevsky (4), the shocks of mortality corresponds to parallel shifts of the term structure of the mortality (from herein TSM). A parallel shift to the term structure of mortality (TSM) is defined as biologically ageing by years in a Gompertzian framework (4). Nevertheless, there is no consistent evidence about the parallel shifts. Furthermore, the virus could be correlated with non-virus mortality in a non-linear way.

In our paper, we were inspired by the empirical evidence on England and Wales that many of those who die from coronavirus would have died anyway in the relatively near future due to their existing frailties or co-morbidities (5). The acceleration conceives the underlying idea according to deaths are “accelerated” ahead of schedule due to COVID-19. From a medical point of view, the susceptibility as COVID-19 is a “newly identified pathogen, there is no known pre-existing immunity in humans. Based on the epidemiologic characteristics observed so far in China, everyone is assumed to be susceptible, although there may be risk factors increasing susceptibility to infection” (1).

Accordingly, in light of these considerations, we focus on a measure of the mortality acceleration, being inclusive of the relative deterioration due to the presence of the comorbidities at COVID-19 diagnosis, the mortality shocks involving parallel shifts of the TSM that are inconsistent with the empirical evidence on the Italian data we analyzed. We model the mortality acceleration by developing a revised Charlson Comorbidity Index in a stochastic setting. The Charlson comorbidity index (6) represents a useful tool for measuring the burden of comorbid disease and its impact on the 10-years mortality projection. The Charlson comorbidity index is based on a proportional hazards model. The proportional hazards model can be usefully implemented to capture the selection effects in the population (7), as observed in the increased mortality rate during the COVID-19 (2).

Nevertheless, we propose a new stochastic formulation of the index, to improve the significant drawbacks of the standard tool. Based on a post-stratification scheme, we obtain an unbiased comorbidity index that varies by age, grounded on the reference population. The new measure appears more suitable and coherent with the mortality acceleration. The layout of the paper is the following. In Section 2 we introduce the general background on the Proportional Hazards Model. Section 3 define the metrics of interest in modeling mortality due to the COVID-19. Section 4 presents the Charlson Comorbidity Index in its standard version. The stochastic formulation of the Charlson Comorbidity Index is proposed in Section 5 as a proxy of the future mortality acceleration. Section 6 shows the numerical results. Section 7 explains the clinical implementation of sCCI. Section 8 concludes.

## 2. Outlook on Life Expectancy Projections

Let us define the natural TSM by the following expression:


(1) $$\begin{array}{r} ln\left[\mu_{x}-\lambda \right]=\ln\left(h\right)+gxx\gg 0 \end{array}$$

where the accidental (Makeham) constant λ≪μ_*x*_ given the natural mortality rate at (chronological) age *x* by


(2) $$\begin{array}{r} \mu_{x}-\lambda =\frac{1}{b}e^{\frac{x-m}{b}}=he^{gx} \end{array}$$

being *m* a modal coefficient, *b* a dispersion coefficient and λ the accidental death rate. The (*m, b*) formulation is used in actuarial finance, where the equivalent relationship holds by implementing the (*h, g*) traditional notation by the demographers and biologists.

A parallel shock to the term structure of mortality can be modeled by *ln*[μ_*x*_−λ] increasing by a constant *v* for all *x* in the Gompertzian age range.

Indeed some authors assume that the total mortality rates during the coronavirus period have been strictly proportional to normal mortality rates, which effectively increase biological ages across the curve, otherwise known as a parallel shift of the (Gompertzian) term structure (4). In (2) as well the stresses and post-pandemic mortality rates are referred to as mortality shocks, by involving parallel shifts of the TSM.

According to (8), we introduce the Mortality Acceleration ${}_{t}^{MA}\vartheta_{x}$ as the spread between the mortality projections:


(3) $${}_{t}^{MA}\vartheta_{x}{=}_{t}^{Acc}q_{x}{-}_{t}^{Base}q_{x}$$

the ${}_{t}^{Acc}q_{x}$ being the accelerated future mortality estimate for individual aged *x* at time *t* obtained by ADM and ${}_{t}^{Base}q_{x}$ the future mortality estimate for individual aged *x* at time *t* obtained by a baseline stochastic model based on the Human Mortality Database (HDM) data.

## 3. General Background on Proportional Hazards Model

In literature, the models to depict the time to occurrence of events are known variously as survival analysis methods, Cox regression, proportional hazards models, duration models ore failure time models, where the incidence, or hazard rate, is the number of new cases of disease per population at-risk per unit time. The hazard function expresses the probability that if a person survives to *t*, they will experience the event in the next instant.

Let denote λ(*t*|*X*_1, *i*_, *X*_2, *i*_, …, *X*_*K, i*_) the hazard function for the *i*−*th* person at time *t*, *i* = 1, 2, ..*n* where *X*_*j, i*_, *j* = 1, 2, .., *K* are the regressors, λ_0_(*t*) is the baseline hazard function at time t, i.e., when *X*_1, *i*_ = 0, *X*_2, *i*_ = 0...*X*_*K, i*_ = 0. Typically the hazard ratio, which corresponds to $\frac{\lambda_{1}\left(t\right)}{\lambda_{0}\left(t\right)}$, represents the relative risk of the event occurring at time *t*.

The logarithm of the hazard function divided by the baseline hazard function at time *t* can be formulated as a linear combination of parameters and regressors:


(4) $$\begin{array}{r} log\left[\frac{\lambda \left(t|X_{1,i},X_{2,i},\ldots ,X_{K,i}\right)}{\lambda_{0}\left(t\right)}\right]={\beta_{1}X}_{1,i}+{\beta_{2}X}_{2,i}+\ldots +\beta_{K}X_{K,i} \end{array}$$

The proportional hazard model can be considered in terms of hazard function at time *t*:


(5) $$\lambda \left(t|X_{1,i},X_{2,i},\ldots ,X_{K,i}\right)=\lambda_{0}(t)e^{\left(\beta_{1}X_{1,i}+\beta_{2}X_{2,i}+\ldots +\beta_{K}X_{K,i}\right)}$$

where is pointed out that the variation in a covariate involves a multiplicative effect on the baseline risk.

This model assumes that the hazard for any individual is a fixed proportion of the hazard for any other individual (*i.e., proportional hazards*). In the graphical analysis, the proportional hazards appear as approximately parallel hazard curves, the hazard curves violating the proportionality assumption when appreciably diverging, converging, or crossing one another.

In the proportional hazards model, the magnitude of the impact of the individual variables and how much the hazard rate is expected to vary as a consequence of changing the individual variables are estimated. For instance, in the analysis of mortality, due to this setting, the relative rates or rate ratios (*RRs*) can be directly calculated, in order to quantify the relative risk of a risk factor on the overall mortality. In other words, the relevant benefit of the proportional hazards consists in its potential to evaluate the effect of a certain risk factor after having incorporated the (confounding) effect of other relevant risk factors on the phenomenon under consideration.

Starting from the concept of relative risk, the Charlson comorbidity index meets the need to combine the changes in mortality risk due both to age and comorbidity in a single index. While statistical models allow us to analyse the effects on mortality risk of the variables considered individually, on the other hand, it is necessary to guarantee a certain sample size to obtain reliable results. However, in most clinical studies, the sample sizes are small and the use of a synthetic index could be a useful tool to ensure the reliability of results (9).

## 4. Standard Charlson Comorbidity Index

The Charlson comorbidity index (6) represents a useful tool for measuring the burden of comorbid disease and its impact on the 10-years mortality projection. The relevance of comorbidity, as expressed by the total burden of chronic diseases, has received surprisingly little attention in the literature, despite its proven ability to predict mortality (10). It is considered a validated method [for instance see (11–14), etc.] of classifying comorbidity to predict short and long-term mortality. In particular, this is a prognostic indicator showing how age and comorbid conditions might alter the risk of mortality. The index assigns weights for specific diseases. A total of 16 comorbidities are included. Specifically, the formula of Charlson Comorbidity probability of 10-years survival can be expressed as follows:


(6) $${}_{10}^{CCI}p_{x}=0.983^{e^{CCI\cdot 0.9}}$$

0.983 being a theoretical low risk 10-years survival probability of an age class from 0 to 50 and *e*^*CCI*·0.9^ representing a proportional hazards model where both comorbidity and age are combined in a scoring system determined according to Hutchinson and Thomas method (15). The lowest score of 0 corresponds to a 98% estimated 10-year survival rate. As the age increases and comorbidities appear, the total score increases and the estimated 10-year survival decreases. The proportional hazards model expresses the relative risk of death due to the presence of adverse prognostic factors, i.e., the comorbidities, the score having assigned by the age-equivalence principle as in (15). For instance, 1 year of codified comorbidity is equivalent to an extra year of age.

Since the CCI is formulated based on the proportional hazard model, it is possible to predict the survival probability for every single patient. However, the acceleration of mortality is an aggregate phenomenon. Furthermore, the acceleration is modeled as a multiplying factor starting from the comorbidity-free population. In this application, the approach proposed by Charlson et al. (6) shows its limits, since the formula is closely linked to the clinical data used to calculate it and cannot be generalized to a specific country population. For this reason, it is necessary to:

- Reformulate the comorbidity index in a such of way that can predict the survival probability at an aggregated age level, using an aggregation criterion not affected by sample bias;
- Define a reliable low-risk population survival probability;
- Reassess the age threshold of the risk-free population and the age equivalence coefficient based on the empirical evidence on the country population data.

Following these steps, we propose a Stochastic Charlson Comorbidity Index (sCCI), used to predict the acceleration of mortality based on a baseline stochastic model, namely a Lee-Carter family model.

## 5. Stochastic Charlson Comorbidity Index: A Proxy of the Mortality Acceleration

Starting from formula 6 we generalize the 10-years survival probability in case of comorbidity as follows:


(7) $${}_{10}^{CCI}p_{x}=\gamma^{e^{CCI\cdot \beta }}$$

where γ is the low-risk population survival probability and β is the age equivalence score. Formula 7 allows to redefine the survival probability through a reparameterization of CCI, γ and β. The procedure for each parameter is described below:

- For CCI the idea is to obtain an index that can synthesize comorbidities aggregated by age. In this sense, it is necessary to consider that clinical data tend to overestimate the incidence of disease in the population. For this reason, a post-stratification strategy is used in the construction of the age index, re-weighing the observations based on the incidence of the disease in the population, using the European Health Interview Survey (EHIS) data (16), an official statistics relating to the incidence of diseases in the main European countries;
- For γ the idea is to adapt the low-risk age threshold to the country population analyzed. For this reason, a sensitivity analysis on age thresholds is performed, to assess the best age threshold for our case;
- For β the idea is to obtain a flexible age equivalence score, using a relative risk ratio computed using the Disability-Free life expectancy, obtained by Sullivan method, and the entire population life expectancy. This ratio represents the difference, in terms of risk of survival, of the comorbidity at a defined age and year.

The reparameterization allows solving the problem of the excessive rigidity of the CCI which, although useful for the analysis of a clinical database, is actually built on the basis of a population of hospitalized patients limited in space and time. This reparameterization process aims to provide a strategy that allows redefining the index in order to be useful for the analysis of aggregate phenomena and of a country population in a certain time and not only on a clinical sample.

### 5.1. Comorbidity Matrix

In order to obtain a CCI aggregated by age, we arrange the vector of individual CCI scores in a matrix age by CCI, which we can call Comorbidity Matrix:


(8) $$\begin{array}{r} \begin{array}{cccc} CCI_{11} & CCI_{12} & \ldots & CCI_{1n}\\ CCI_{21} & CCI_{22} & \ldots & CCI_{2n}\\ \vdots & \vdots & \ddots & \vdots \\ CCI_{\omega 1} & CCI_{\omega 2} & \ldots & CCI_{\omega n} \end{array} \end{array}$$

where we have for each age (by row) different score of comorbidity, i.e., CCI, since each age has more than one patient with a different score. To obtain a unique measure for each age class, we calculate an average by row. In particular, we develop a weighted average, since the data of hospitalized patients overrepresent the presence of comorbidities in the case of COVID-19 infection, with a consequent overestimation of survival probability in the event of comorbidity. Our intuition of calculating the weights relies on the logic of the survey post-stratification strategy, so that we attribute a weight to our sample affected by comorbidities, based on the proportion of the individuals affected by comorbidities on the total exposures to the risk of death. Post-stratification is a very common practice in survey analysis, that allows avoiding the selection bias and the response bias of the estimators (17, 18). Similarly, we consider CCI as an estimator of the comorbidity of a country's population and it is necessary to avoid the selection bias of the use of a clinical dataset instead of a random stratified sample.

Since a list of the population of CCI is not available, but only the presence of a certain disease is, the weights are inserted within the index, in such a way as to obtain a score weighted by the incidence of the population of a given disease:


(9) $$\begin{array}{r} \bar{{CCI}_{x}}=\frac{1}{n_{x}}\overset{K}{\underset{k=1}{\sum }}\overset{n_{x}}{\underset{i=1}{\sum }}W_{k}\;{score}_{k}^{x_{i}}\forall x=1,..,\omega \end{array}$$

where:

*CCI*_*x*_ is the weighted CCI for age *x*;

*W*_*k*_ is disease weight *k* measured as the incidence on the total of comorbid individuals according to the EHIS survey;

${\text{score}}_{k}^{x_{i}}$ comorbidity score according to the CCI original formula for each individual *i* of *x*;

*n*_*x*_ number of individuals of age *x*.

In this case, the stratified sampling idea is used to calculate the score at age *x*, which is given by the score for the severity of the disease according to the definition of the CCI and the weight given by the proportion of the incidence of that disease on the total of the comorbid population:


(10) $$\begin{array}{r} W_{k}=\frac{N_{k}}{N} \end{array}$$

where:

*N*_*k*_ is the number of people at age *x* that present the disease *k*. *N* is the number of people with almost one disease.

### 5.2. Low-Risk Population Sensitivity

As previously noted, the low-risk population thresholds are obtained based on a study by Charlson et al. (6). The low-risk population survival probability is computed on a cohort of 604 patients of the New York Hospital in 1 month of 1984. Since this is not a random sample, but a clinical dataset limited in space and time, it could be necessary to redefine the low-risk population and the relative survival probability in such a way that is adaptable to a different population in time and space. In this sense, a sensitivity analysis is carried out on the age threshold to assess whether a threshold of 50 years can also be valid for populations other than the clinical dataset used to define CCI. Table 1 shows the different scores by age used to define the alternative low-risk populations in CCI estimation.

**Table 1:** CCI score according to low-risk age threshold.

**Age threshold**	**Age class**	**Score**
20	0–20	0
	20–30	1
	30–40	2
	40–50	3
	50–60	4
	60–70	5
	70–80	6
	80 and more	7
30	0–30	0
	30–40	1
	40–50	2
	50–60	3
	60–70	4
	70–80	5
	80 and more	6
40	0–40	0
	40–50	1
	50–60	2
	60–70	3
	70–80	4
	80 and more	5
50	0–50	0
	50–60	1
	60–70	2
	70–80	3
	80 and more	4

### 5.3. Age Equivalence Score

Following the same principles of the low-risk population survival probability redefinition, we reconsider the age equivalence score, computing the RRs of combined comorbidity and age using the survival probabilities of the life table. Following the Sullivan method (19), we define the RRs as the ratio between the entire population survival probability and the disability-free survival probability. The estimation of the disability-free survival probability follows the idea of the construction of the disability-free life expectancy, computing the disability-free survival as follows:


(11) $${}_{t}^{DF}L_{x}={\;}_{t}L_{x}\cdot (1-\pi_{x})$$

where:

__*t*_*Lx*_ is the survival in the mortality table. π_*x*_ is the proportion of individuals with disabilities in the population of the mortality table.

the survival probability is computed as the ratio between the survival at age *x* and the survivals at age *x*+1:


(12) $${}_{t}^{DF}l_{x}=\;\frac{{}_{t}^{DF}L_{x}}{{}_{t}^{DF}L_{x+1}}$$

Finally, the *RR*s are defined as:


(13) $$RR=\;\frac{{}_{t}l_{x}}{{}_{t}^{DF}l_{x}}$$

## 6. Numerical Applications

In this section, we investigate the presence of mortality acceleration in the Italian population due to COVID-19 infection, using an “empirical” approach, based on observed data and a “modeling” approach, estimating the expected acceleration based on a stochastic CCI. To obtain the considered result we proceed by the following steps: in first, the baseline mortality using a Lee-Carter model (20) of all-aged aggregated Italian population has been estimated, the second step consists in estimating the acceleration function as in Cairns et al. (5), using a data-driven approach and considering two different scenarios: the former is based on ISS data, relating to the entire population, the latter is based on SIIA data, relating to a hospitalized population in 26 hospitals and centers, located in 13 regions in Italy (21). The use of both datasets allows focusing the analysis on two different aspects. ISS dataset reports weekly the number of death by COVID-19 in Italy by gender and age, but provide no information about comorbidities. On the contrary, the SIIA dataset is based on a subpopulation of hospitalized people by COVID-19. The information is classified by age, gender, and co-morbidities. In this sense, the analysis focused firstly on the acceleration effects due to age and then on the acceleration due to comorbidities. The final step is the estimation of the Stochastic CCI, whose procedure will be described in detail below.

### 6.1. Estimation of Observed Acceleration

The estimation of the baseline scenario is obtained using a Lee-Carter model using the all-aged aggregated Italian population, both for male and female, estimated from 1950 to 2017, using the Human Mortality Database (2020) data. The Lee-Carter model (22) expresses the improvements in the mortality trend and it allows to capture the changes in the mortality trend. The data are easily implemented, capture the correct description of the phenomenon and consequently highlight the changes in this trend.

The COVID-19 deaths *d*_*c*_(*x, t*) are obtained starting from the projection of all causes *d*_*a*_(*x, t*), obtained by baseline scenario function. Since data about COVID-19 deaths at age *x* are not directly available, the procedure described below is followed:

- Estimation of amplitude α(*x*) as the proportion of the COVID-19 death on all causes of death. The number of COVID-19 deaths is estimated using the two scenarios given by ISS and SIIA datasets. For each database, the sample proportion is computed and multiplied by the number of deaths at 30^th^ November 2020, obtaining an estimation of the number of COVID-19 deaths by age. Then the proportion of deaths in the population is computed using the mortality for all causes data by age groups of 10 years published by ISTAT;
- Estimation of the reach ρ(*x*) as the life expectancy reduction by age groups due to COVID-19 mortality, as follows: the life expectancy at age *x* for 2021 is estimated and the year losses are estimated as the product of the weight of COVID-19 deaths by age, that is the proportion of deaths at age *x* on the total of COVID-19 deaths and the life expectancy estimated in the baseline model;
- Finally, π(*x, t*) is estimated, in the form on negative exponential function, as defined by (5).

Table 2 shows the estimated values of amplitude α(*x*), reach ρ(*x*) and acceleration π(*x*) for the two scenarios, computed from 1^st^ January to 30^th^ November 2020, both for SIIA and ISS scenarios:

**Table 2:** Estimation of amplitude, reach, and acceleration.

**Age**	**All causes**	**Life**	**SIIA**			**ISS**		

**class**	**of death**	**expectancy**	**Amplitude**	**Reach**	**Acceleration**	**Amplitude**	**Reach**	**Acceleration**
0–9	906	78.92	0.000	0.473	0.000	0.006	0.011	0.546
10–19	958	68.98	0.272	1.195	0.206	0.005	0.004	0.990
20–29	2,311	59.15	0.352	2.976	0.092	0.015	0.025	0.602
30–39	3,890	49.35	0.364	5.311	0.044	0.037	0.099	0.376
40–49	11,564	39.66	0.167	6.898	0.014	0.052	0.357	0.140
50–59	29,221	30.28	0.318	6.226	0.030	0.069	1.051	0.061
60–69	61,998	21.50	0.353	5.388	0.042	0.095	2.135	0.037
70–79	132,227	13.62	0.143	1.943	0.062	0.108	3.481	0.023
80–89	244,434	7.29	0.014	0.296	0.045	0.087	2.993	0.023
90+	146,379	3.34	0.003	0.018	0.174	0.060	0.628	0.091

As can Table 2 shows, the estimates based on the SIIA and ISS scenarios differ evidently, in the function of the age group.

The amplitude is higher for ages up to 69 years in the SIIA scenario compared to the ISS scenario, similar for ages from 70 to 90 years and lower for the 90+ age group. In other words, the SIIA scenario gives more emphasis on not extreme age group than the ISS scenario. This aspect could depend on the different nature of data, since, as specified previously, SIIA data are based on COVID-19 hospitalizations in presence of comorbidities, that aggravate the clinical course and therefore the possible death. In this sense, the SIIA scenario highlighted the effects of co-morbidities on COVID-19 mortality.

The reach is higher for ages up to 69 years in the SIIA scenario than in the ISS scenario, with a loss of life expectancy of up to about 5–7 years in the age groups between 30 and 69 which, in this case, are the most affected group. On the contrary, in the ISS scenario, the most affected age groups are those between 60 and 89 years, with a loss of life expectancy between 2 and 3 years. Also in this case the difference depends not only on the distribution by different ages of the two databases on which scenarios are based but also on the different distribution of frailties. The greater fragility determines a greater loss of years of life expectancy due to the COVID-19 infection.

**Figure 1:**
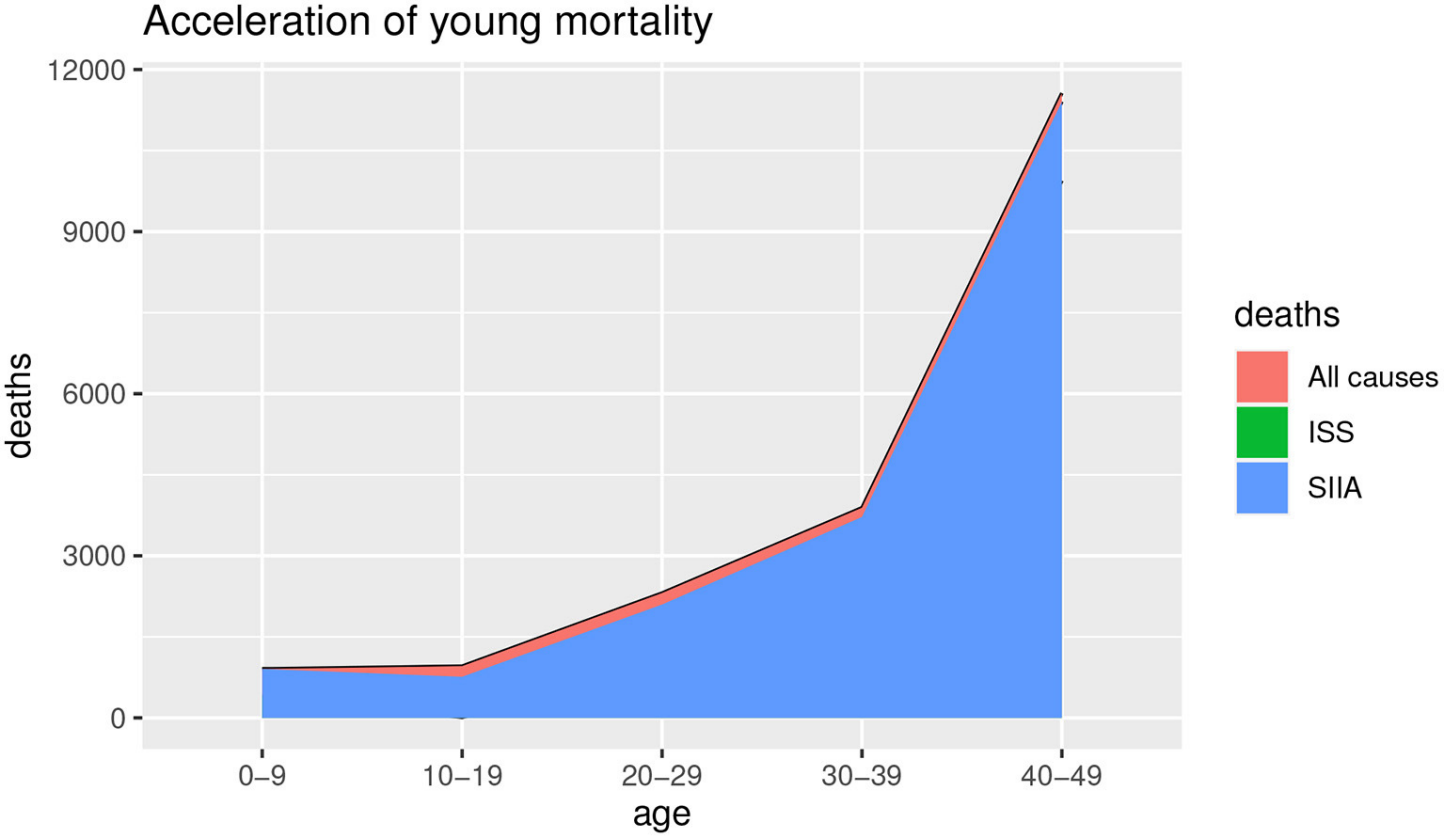
Estimation of all causes of deaths and non-COVID-19 deaths. Young ages.

**Figure 2:**
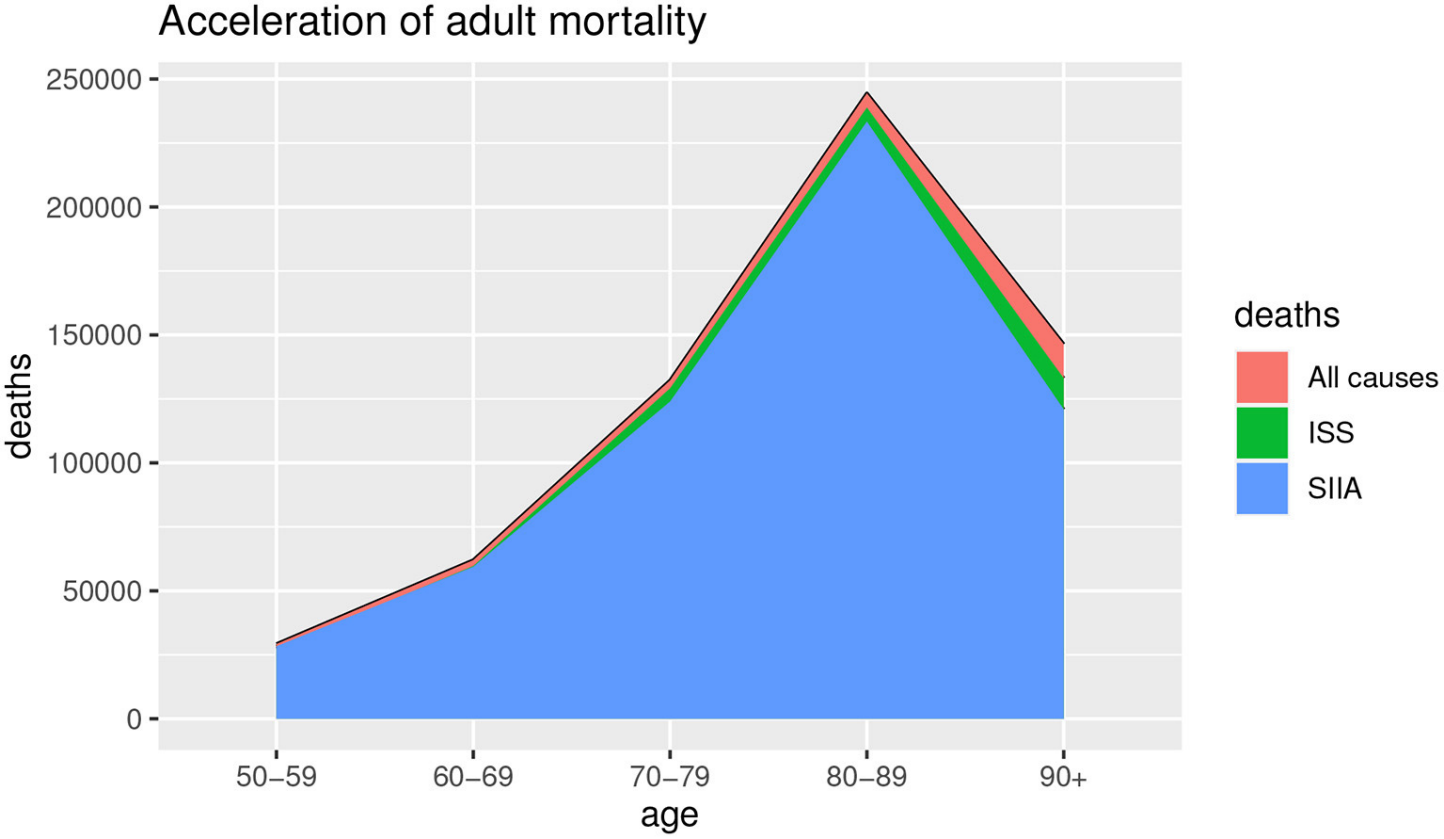
Estimation of all causes of deaths and non-COVID-19 deaths. Adult ages.

Figures 1, 2 show the deaths for all causes *d*_*a*_(*x, t*), obtained by baseline scenario and the non-COVID-19 deaths *d*_*s*_(*x, t*) estimated on the data, based on ISS and SIIA scenarios, as the difference between the *d*_*a*_(*x, t*) and the COVID-19 deaths *d*_*c*_(*x, t*), obtained by acceleration function π(*x*). In this way, acceleration death area is highlighted. In particular, Figure 1 shows the effects of acceleration for young ages (up to 50 years) and Figure 2 the effects of acceleration of adult ages (over 50 years).

As we can observe from Figures 1, 2, the acceleration factor is different according to age groups and considered scenarios. For the younger ages, although there is an acceleration, the differences between the two scenarios are negligible. On the contrary, for adult ages, for both scenarios, there is an acceleration, but the ISS scenario is less pessimistic than SIIA, with a higher non-COVID-19 death (green area vs. blue area). This aspect underlines the importance of distinguishing between fragile and non-fragile populations in the context of the analysis of the effect of COVID-19 mortality.

**Figure 3:**
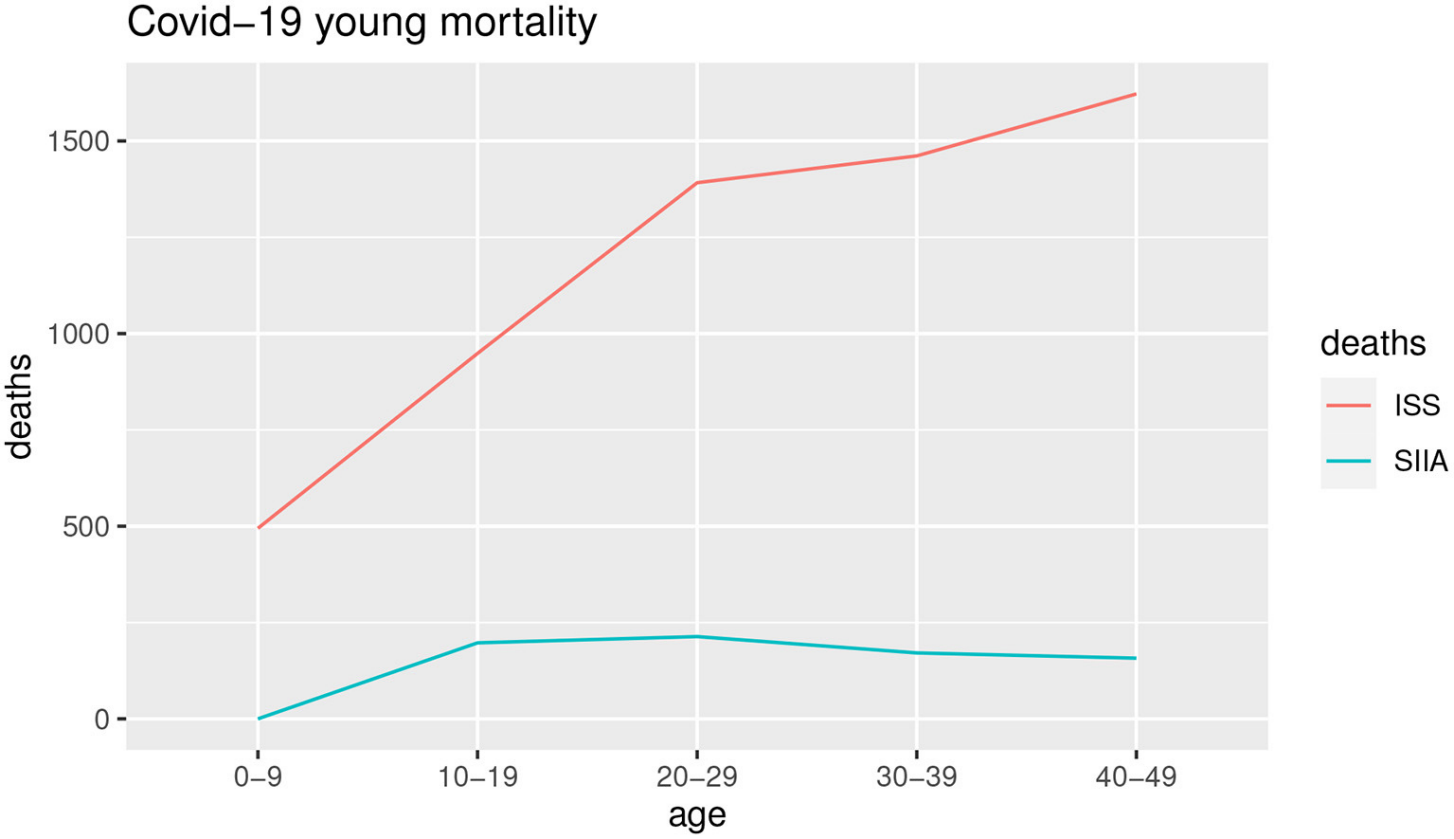
Estimation of non-COVID-19 deaths. Young ages.

**Figure 4:**
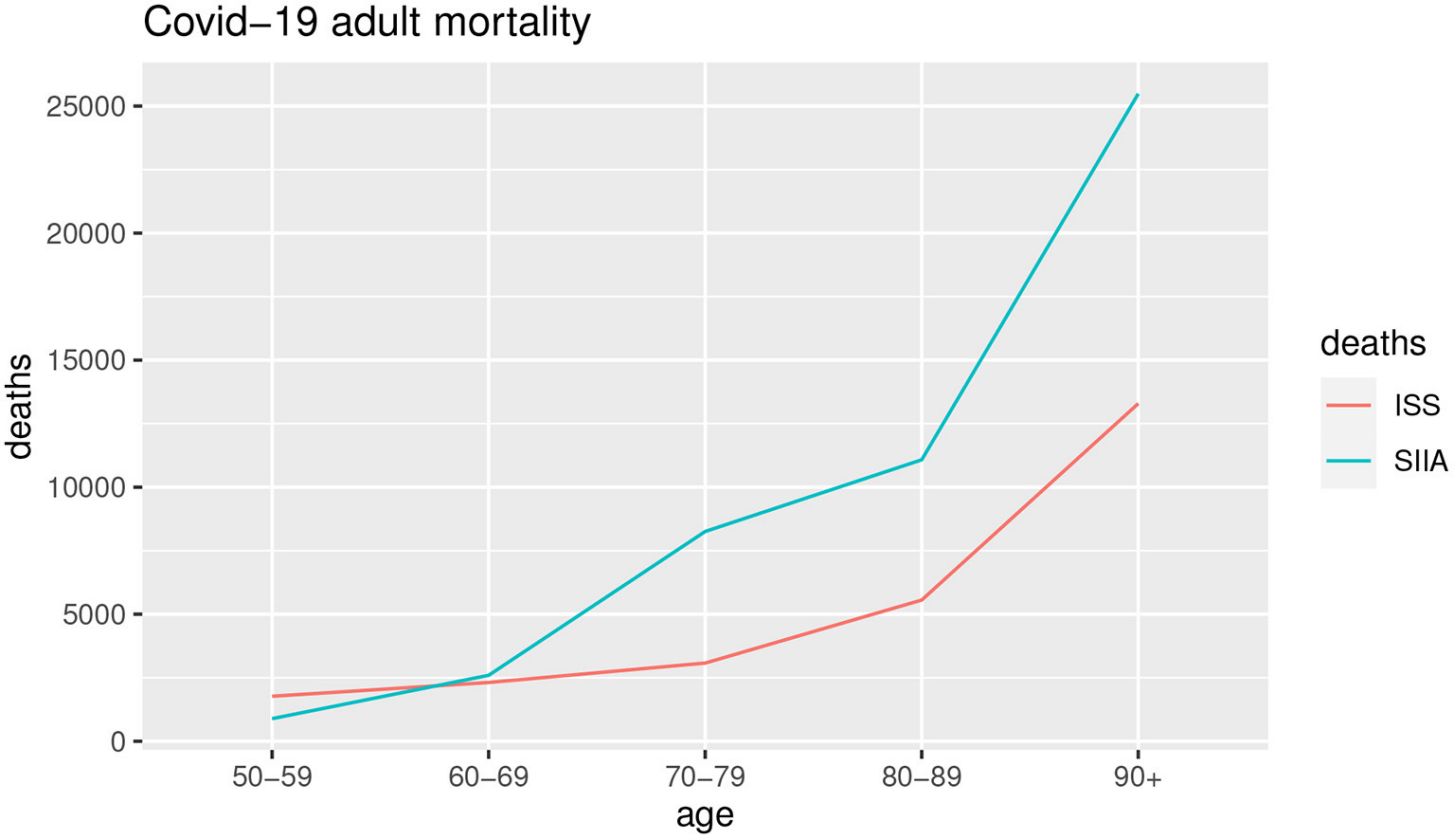
Estimation of non-COVID-19 deaths. Adult ages.

Figures 3, 4 show the COVID-19 deaths *d*_*c*_(*x, t*) obtained for young and adult ages both for SIIA and ISS scenarios.

As shown by Figures 3, 4, the COVID-19 mortality for young ages is greater than the ISS scenario with respect to SIIA, while, the opposite is observed for adult ages, in particular over 60 years old. As highlighted previously, ISS data tends to be more optimistic for adult ages, due to take not into account the comorbidity conditions of the infected.

### 6.2. Estimation of Expected Acceleration

In order to obtain a measure of expected accelerated deaths, that is the survival probability on the basis of comorbidity population, we propose an alternative measure of CCI, called stochastic CCI, obtained with the following procedure:

- Estimation of an age-aggregated CCI, obtained by the weighted mean of individual CCI using a post-stratification weighting scheme;
- Definition of a flexible low-risk population survival probability, calibrating the 10-years survival probability on the basis of the age threshold to assign the zero score and the estimation of the Disability-Free survival probability, as an indicator depending on both age and year;
- Definition of a flexible age equivalence score, using a relative risk ratio on the basis of Disability-Free life expectancy and the entire population life expectancy, as the difference, in terms of risk of survival, of the comorbidity at a defined age and year.

CCI is usually measured for every single patient, but the interest of the analysis is on the aggregated population by age groups. For this reason, it is necessary to synthesize it. The simplest way to synthesize data by age groups is the mean, but taking into account that the proportions of the co-morbidities in the SIIA dataset could not be representative of the entire Italian population, we use a weighted mean, as a post-stratification of the sample.

The first step is the reorganization of the SIIA database by age, obtaining a matrix that we can call Comorbidity Matrix, based on the CCI scores:


(14) $$\begin{array}{r} \begin{array}{cccc} CCI_{11} & CCI_{12} & \ldots & CCI_{1n}\\ CCI_{21} & CCI_{22} & \ldots & CCI_{2n}\\ \vdots & \vdots & \ddots & \vdots \\ CCI_{\omega 1} & CCI_{\omega 2} & \ldots & CCI_{\omega n} \end{array} \end{array}$$

The following step is to compute the weighted mean to obtain a weighted CCI by age. Weights are obtained from European Health Interview Survey (EHIS) data, whose age groups are used. To observe if the use of the weighted average allows obtaining more reliable results, weighted CCI by age is compared with the unweighted CCI by age in Table 3.

**Table 3:** Weighted vs. unweighted CCI.

**Age**	**wCCI**	**uwCCI**
15–17	0.001	0.129
18–19	0.002	0.625
20–24	0.001	0.356
25–34	0.005	0.542
35–44	0.025	0.719
45–54	0.503	1.545
55–59	1.207	2.415
60–64	2.106	3.464
65–74	2.759	4.209
75+	3.868	5.592

As shown in Table 3, the use of a weighting scheme greatly reduces the age score. This is an important aspect because it allows the probability of survival in the event of co-morbidities to be estimated more reliably than using a sample in which the co-morbidities are overrepresented.

**Figure 5:**
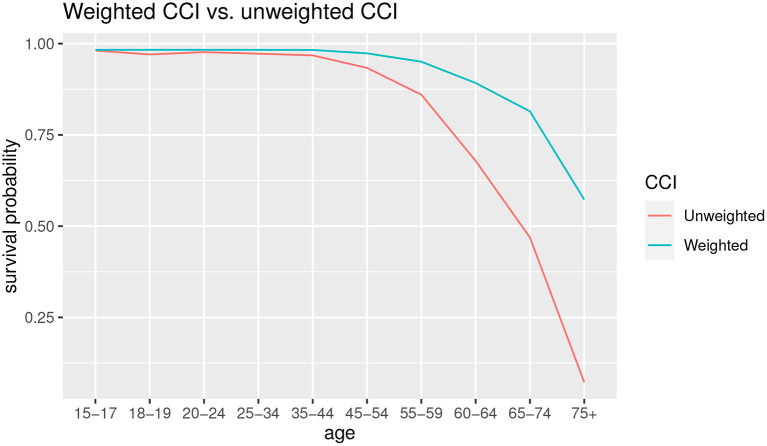
Survival probability with weighted CCI and unweighted CCI.

The effects on the estimation of the probability of survival can be seen in Figure 5.

As can we observe from Figure 5, for the younger ages there are few differences in survival probabilities, instead the gap widens starting from the 45–54 age group, with an increasingly lower probability of survival in the case of unweighted CCI. In the latter group, 75+, the probability of survival in case of co-morbidity using the unweighted CCI is almost zero, while in the case of the weighted CCI it is about 0.5.

**Figure 6:**
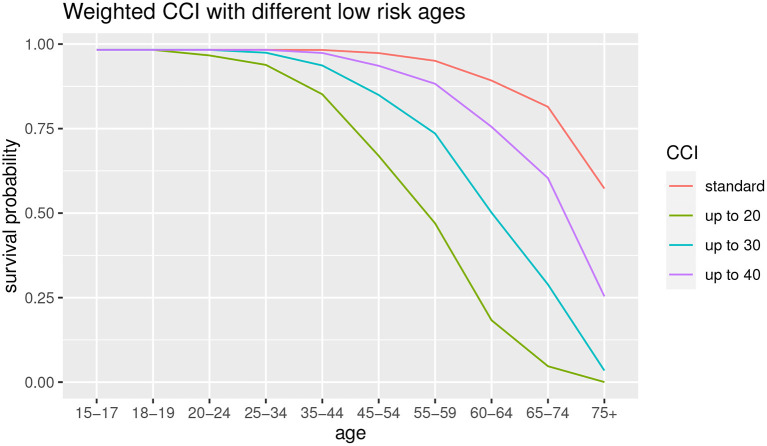
Survival probability with different age thresholds.

Subsequently, a robustness analysis is carried out relating to the choice of the low-risk age group. In the standard CCI the low-risk age is up to 50. We observe the difference among the use of different age thresholds, that is up to 20, up to 30 and up to 40, in order to observe the differences in terms of survival probabilities. The results are shown in Figure 6.

As can we observe from Figure 6, the lower is the low-risk age group, the lower is the survival probability for all the age groups. This result suggests that the age threshold of 50 years (original CCI, in red) is not robust with respect to the survival probability estimation. We can also observe that the 20 years old and 30 years old thresholds show a functional form different from other survival probability functions. It suggests that it is preferable to take into account a lower age as low-risk population but not excessively, to avoid the risk of underestimation for the elder ages. For this reason, we consider a 40-year-old threshold.

Then, we estimate a Disability-Free survival probability by age as an alternative baseline to propose. Following the Sullivan method (19), we estimate the survival probability from a life table. In our case, we compute a projected life table from RH model and calculate the survival probability as the ratio of person-years of life at age *x*+1 weighted by the proportion of disability-free people at the age *x*+1 and the person-years of life at age x weighted by the proportion of disability-free people at the age *x*. Results are shown in Table 4.

**Table 4:** Disability-free survival probability.

**Age**	**Survivals DF**	**Probability DF**
15–17	91207.39	–
18–19	90540.2	0.9927
20–24	89661.82	0.9903
25–34	89510.3	0.9983
35–44	83170.82	0.9292
45–54	72536.36	0.8721
55–59	61974.57	0.8544
60–64	57254.69	0.9238
65–74	48255.94	0.8428
75+	28605.37	0.5928

As can we observe from Table 4, disability-free survival probability is higher for the younger ages. In the age groups 45–54 and 55–59 the survival probability is lower than the group of 60–64. This aspect can be interpreted as a greater onset of co-morbidities in those age groups with respect to 60–64 one. In this sense, reaching a certain age group without co-morbidities ensures greater longevity than in the younger groups.

The final step is to estimate the age equivalence score. Following the idea of (15), and (9), a relative risk of survival probability between Disability-Free and the frail population is calculated. Since the relative risk is the ratio of the incidence of death considering the presence of a particular condition (COVID-19 comorbidities in this case) and the incidence of death in its absence, we estimate the relative risk using the Disability and Disability-free deaths. Results are shown in Table 5.

**Table 5:** Relative risk of death between Disability-Free and frail populations.

**Age**	**Relative risk**
15–17	–
18–19	0.9928
20–24	0.9904
25–34	0.9985
35–44	0.9295
45–54	0.8734
55–59	0.8574
60–64	0.9292
65–74	0.8535
75+	0.7033

As shown in Table 5, the relative risk is higher for the frail population with respect to disability-free population, and higher for higher age groups up to a certain age and then decrease slowly. In particular, the maximum value is for the ages 60–64, and it remains quite constant for the ages 65–75 and lower for 75+ age group.

Using the parts we previously showed, we calculate the theoretical survival probability using the CCI formula (6), with the components we previously estimated, that is:


(15) $${}_{x}^{DF}p_{t}e^{{\text{wCCI}}_{40}RR}$$

where:

${}_{x}^{DF}p_{t}$ is the disability-free survival probability;

wCCI_40_ is the weighted CCI, using 40 years as the threshold of the low-risk population;

*RR* is the relative risk of survival in case of co-morbidities.

**Figure 7:**
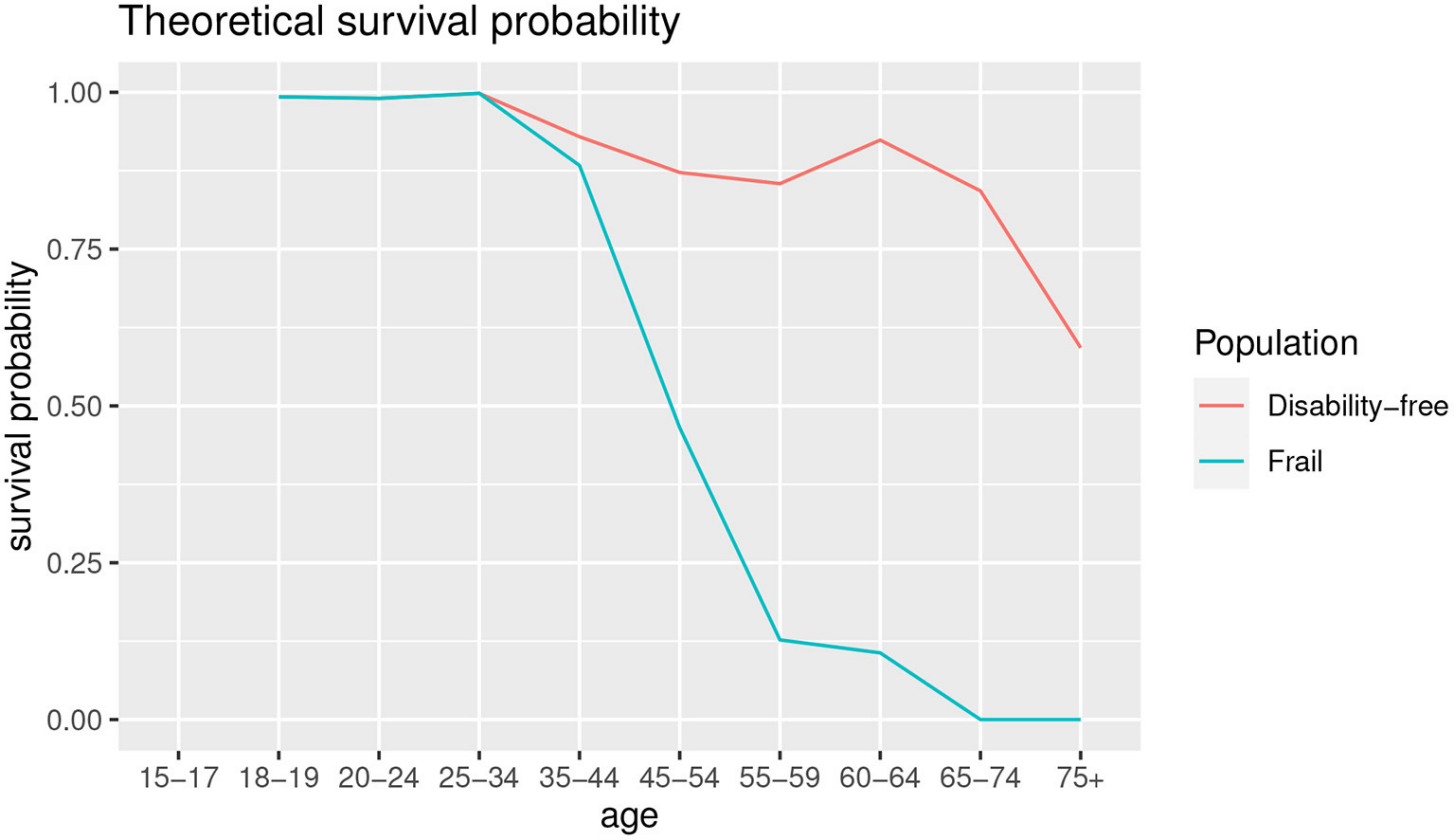
Theoretical survival probability.

Figure 7 shows the theoretical survival probability compared to disability-free survival probability shown in Table 4.

As shown in Figure 7, the comorbidity is an important driver of survival probability. Except for the age groups up to 25–34, the survival probability in presence of co-morbidities is lower than disability-free population, and the gap is wider as the age increases, with a slight improvement for the age 60–64 for both the survival functions.

### 6.3. A Compact Discussion: From the Data to Results

We represent the CCI indicator as a population comorbidity measure that varies by age based on clinical information collected by hospitalized patients. The clinical data show lower survival probabilities by definition, in comparison with an aggregated national population, showing a sort of the actuarial adverse selection (23). To overcome this phenomenon which determines biased estimates due to the overestimation of the actual prevalence of the disability in the population, we propose a post-stratification scheme that allows for a sample re-weighting. In particular, we aggregate the CCI by this approach based on the European Health Interview Survey. Accordingly, we summarize a CCI based on age, by obtaining an unbiased and consistent score coherently with the reference population.

The weighted CCI by the prevalence of the disabilities in the Italian population shows a lower score by age concerning the standard CCI. This implies the opportunity of considering the CCI as an aggregate index of population comorbidity by age. Furthermore, using official data on disability incidence, the low-risk population survival probability and the relative risk of survival between disability-free and disability can be obtained, which are used to replace the coefficients of the CCI standard, to adapt the survival probability in case of co-morbidities to the Italian framework. As a result, it would be more appropriate to lower the low-risk population age threshold to 40 years, and the relative risk increases as the age increase up to 75 years. It is also noted that survival for the disability-free population tends to be higher for some older age groups, such as 60–64 and 65–74 than others.

### 6.4. Strengths and Potential Drawbacks

The potential drawback related to our proposal consists in assigning the age threshold for computing the low risk population survival probability. The original threshold posed by the standard CCI at level of 50 age represent an unreliable value since, as intuitively expected, as the age increases lower survival probabilities we estimate. So that, for avoiding the threshold inconsistency in the standard CCI setting, we modify the thresholds as described in Section 6.2, by means of an heuristic approach which gradually decreases the value of the threshold according to a sensitivity analysis. From the sensitivity analysis a potential inconvenience emerges: as decreases the age value of the threshold, being theoretically consistent with a low risk population, a CCI score increase arises due to the age effect. In other terms, for higher ages than the threshold, as the age value threshold decreases, the CCI score make worse, i.e., increases due to the contribution of the age to the CCI score (a sort of age effect). The balance between the age effect and the contribution of comorbidities on the estimation of the CCI score value, we call comorbidity effect, can be a problematic aspect to take into account in choosing the “right” age threshold. In the future research we are going to develop an optimization algorithm for minimizing the error of the procedure.

The advantages of the new proposal of the CCI relies in the stochastic environment of the calculus instead of a poor deterministic approach that makes more reliable the estimates and in the clinical implementation of this accurate index. The clinical computation has been widely detailed in the following section, by stressing the support for medical problems in processing information recorded by medical data.

## 7. Clinical Implementation of sCCI

The sCCI can have great clinical utility and support medical practical problems in collecting and analyzing the medical data, to gain transferable insights in medical research.

In particular, based on the clinical picture of a patient, the sCCI index allows calculating the specific risk of mortality by calibrating the scores according to the comorbidity and longevity of the population he/she belongs to (reference population). In this section, we disentangle the determinants for the calculation of the sCCI as in formula 7, to show the index implementation for clinical evaluations by an example of the case study.

The sCCI consists of the following components:

1. β;
2. γ;
3. Comorbidity score;
4. Age score.

β and γ represent idiosyncratic factors specifically related to the reference population. In this paper, as explained in Section 5, we re-calibrate the value of the parameters of the Italian population.

**Figure 8:**
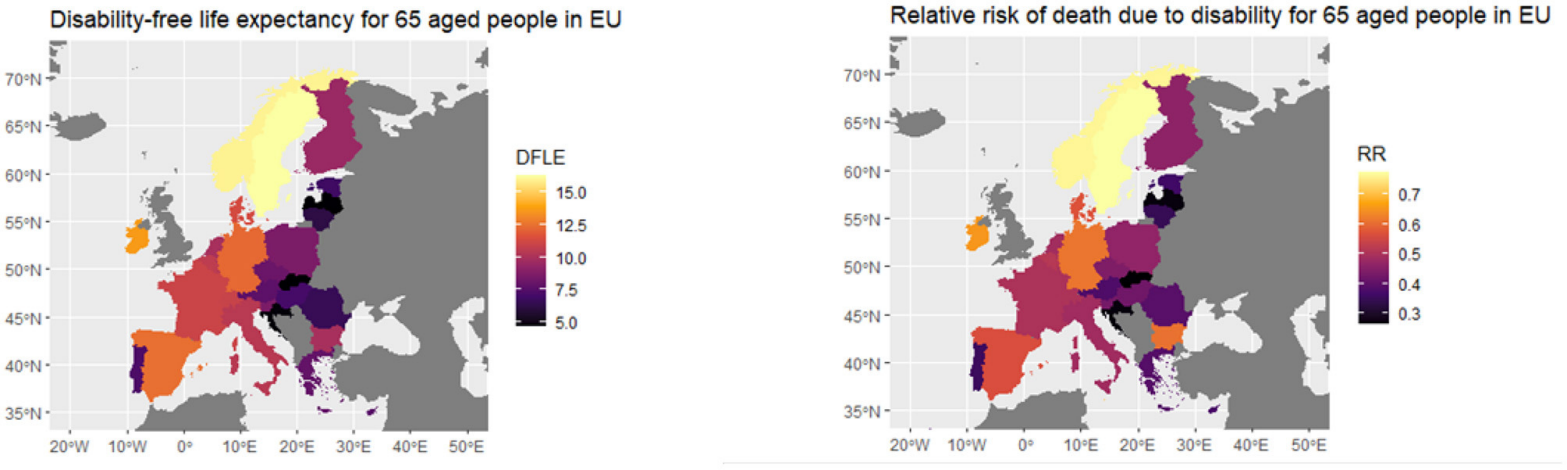
Disability-free survival probability and relative risk for 65yo in EU. Eurostat.

For sake of clarity, in the following, we offer a picture of the specific parameters for each European population that have to be implemented for obtaining the idiosyncratic factors beta and gamma. Indeed in Figure 8, we provide the different values of the disability-free life expectancy (DFLE) for individuals aged 65, for geographical areas in Europe (24) and relative risk measured by years lost in the life expectancy due to disability (RR) we compute based on the DFLE.

As Figure 8 shows, Eastern Europe countries, Slovakia, Croatia and Portugal have a lower DFLE than the others, while the Scandinavian countries have the highest values, followed by Germany, Spain and Ireland. RR, on the other hand, is the highest for the Scandinavian countries, followed by Germany, Ireland and Hungary, while it is the lowest for the Baltic countries, Portugal, Slovakia and Croatia. In general, it can be thought that the higher the life expectancy without disability, the lower the relative risk.

The comorbidity score and age score are the competing determinants of the standard CCI, as proposed in (6). The comorbidities score depends on the clinical sample observed on *N* patients and it is computed as in (6). As regards the age score, we propose to modify the calculation basis, according to the following considerations. We change the threshold or maximum value of the age class which constitutes the basis of the calculation. In particular, we adopt the age class 0–20 instead of 0–40, intuitively based on the idea of lower mortality risk as the age decreases.

Hence, let us consider an example of an Italian patient aged 50 with hypertension, diabetes, and chronic rheumatic diseases. Based on Table 1, the age score corresponds to 4. According to (6), the comorbidity score is 3. The CCI comes from the sum of the age score and comorbidity one. Accordingly, in this case it amounts to 7. Being the β and γ parameters respectively 0.8721 and 0.8734 as we computed for the Italian population by the HMD, the sCCI for the patient under consideration corresponds to 0.4331.

## 8. Concluding Remarks

The empirical evidence in medical and actuarial literature shows that many of those who die from coronavirus would have died anyway in the relatively near future due to their existing frailties or co-morbidities (5, 8, 25–27). The underlying idea according to deaths is “accelerated” ahead of schedule due to COVID-19 representing a mortality acceleration. We focus on the future evolution of the mortality acceleration due to the COVID-19 by setting up the Charlson Comorbidity Index in a stochastic setting. We propose a new stochastic formulation of the index, to improve the significant drawbacks of the standard tool. Based on a post-stratification scheme, we obtain an unbiased comorbidity index that varies by age, grounded on the reference population, that represents a good proxy of the future evolution of the mortality acceleration.

In this perspective, it is possible to consider the stochastic CCI as an index that measures the age comorbidity of a population and can be adapted in time and space through the use of easily available data, such as the incidence of disability in the population. Moreover, in a dynamic perspective, through the evolution of the probability of survival, the stochastic CCI can be used to evaluate the decay of the population according to the incidence of disability over time. In other words, we can consider extending the use of CII from the clinical field to a more general one on the analysis of longevity considering the presence of co-morbidities in the population.

In our work, we study on national all-causes-of death mortality aggregated by geographical area and comprehensively including in-hospital and out-of-the hospital mortality that has been adjusted by means of some clinical scores. Nevertheless, in the future research we will focus on widening the predictors for better projecting the mortality phenomenon by selecting other relevant diseases such as atrial fibrillation or HR and so on, by selecting the most important features according the variable importance algorithm artificial intelligence-based. For instance, chronic kidney disease (CKD), incidence of acute kidney injury (AKI) and atrial fibrillation (AF) have been shown to represent comorbidities associated with reduced survival in patients hospitalized for COVID-19 disease. The role of the comorbidities under consideration particularly on in-hospital mortality has been well-documented in some studies as in (28, 29) and others. Accordingly, it could be useful to adjust the stochastic CCI according to a correction factor based on these risk factors, for obtaining a more reliable projections.

Further research we will focus on the long-run mortality projections, to detect the systematic nature of the acceleration.

## Data Availability Statement

All the data used during this study are openly available from different public datasets. Data relating to Italian mortality rates are available from Human Mortality Database at: https://www.mortality.org/Country/Country?cntr=ITA; Data relating to Covid-19 mortality in Italy are available from ISTAT at: https://www.istat.it/it/archivio/240401; Data relating to comorbidities incidence in Italy are available from ISTAT at: https://www.istat.it/it/archivio/210553; Clinical data are available in the repository created by Guido Iaccarino et al. ClinicalTrials.gov; Unique identifier: NCT04331574 as cited in Iaccarino et al. (21).

## Author Contributions

GI and VD'A contributed to conception and design of the study and wrote the first draft of the manuscript. MC and VD'A organized the database. MC performed the statistical analysis. All authors wrote sections of the manuscript, contributed to manuscript revision, read, and approved the submitted version.

## Conflict of Interest

The authors declare that the research was conducted in the absence of any commercial or financial relationships that could be construed as a potential conflict of interest.

## Publisher's Note

All claims expressed in this article are solely those of the authors and do not necessarily represent those of their affiliated organizations, or those of the publisher, the editors and the reviewers. Any product that may be evaluated in this article, or claim that may be made by its manufacturer, is not guaranteed or endorsed by the publisher.
